# Influence of Functional Magnetic Resonance Imaging Data Preprocessing Pipelines on the Accuracy of Schizophrenia Classification Using Machine Learning Methods

**DOI:** 10.17691/stm2026.18.3.02

**Published:** 2026-06-30

**Authors:** A.A. Poyda, V.A. Orlov, A.D. Zhemchuzhnikov, S.O. Kozlov, S.I. Kartashov, L.V. Bravve, M.A. Kaydan, G.P. Kostyuk

**Affiliations:** 1 PhD, Leading Researcher; National Research Center “Kurchatov Institute”, 1 Akademika Kurchatova Square, Moscow, 123182, Russia; 2 PhD, Senior Researcher; National Research Center “Kurchatov Institute”, 1 Akademika Kurchatova Square, Moscow, 123182, Russia; 3 Research Laboratory Assistant; National Research Center “Kurchatov Institute”, 1 Akademika Kurchatova Square, Moscow, 123182, Russia; 4 Junior Researcher; National Research Center “Kurchatov Institute”, 1 Akademika Kurchatova Square, Moscow, 123182, Russia; 5 Researcher; National Research Center “Kurchatov Institute”, 1 Akademika Kurchatova Square, Moscow, 123182, Russia; 6 MD, Junior Researcher, Psychiatrist; Alekseev Psychiatric Clinical Hospital No.1, Moscow Department of Health, 2 Zagorodnoe Shosse, Moscow, 117152, Russia; 7 MD, PhD, Senior Researcher, Psychiatrist; Alekseev Psychiatric Clinical Hospital No.1, Moscow Department of Health, 2 Zagorodnoe Shosse, Moscow, 117152, Russia; 8 MD, DSc, Professor, Chief Physician; Alekseev Psychiatric Clinical Hospital No.1, Moscow Department of Health, 2 Zagorodnoe Shosse, Moscow, 117152, Russia; Scientific Director of the Scientific and Clinical Research Center for Neuropsychiatry; Alekseev Psychiatric Clinical Hospital No.1, Moscow Department of Health, 2 Zagorodnoe Shosse, Moscow, 117152, Russia; Head of the Department of Mental Health; Lomonosov Moscow State University, 1 Leninskie Gory, Moscow, 119991, Russia; Professor, Department of Psychiatry; Russian Biotechnological University, 11 Volokolamskoe Shosse, Moscow, 125080, Russia; Professor, Department of Psychiatry and Psychosomatics; I.M. Sechenov First Moscow State Medical University (Sechenov University), 8/2 Trubetskaya St., Moscow, 119991, Russia

**Keywords:** schizophrenia classification, data preprocessing, fMRI, machine learning methods

## Abstract

**The aim of the study** was to analyze the influence of various pipelines for preprocessing raw functional magnetic resonance imaging (fMRI) data on the accuracy of classification of subjects into schizophrenia patients and healthy controls using machine learning methods, and to give recommendations for optimizing the data preprocessing pipeline for this task.

**Materials and Methods:**

The study used fMRI data from 72 subjects acquired on a Siemens Magnetom Verio 3T MRI scanner (Siemens Healthineers, Germany) at the National Research Centre “Kurchatov Institute”. Seven different preprocessing pipelines were used on each dataset. Feature vectors for classification were constructed from each preprocessed dataset. We applied the following three algorithms for feature vector construction: ReHo, FCM, and FHR. Classification was performed using 15 machine learning methods from the scikit-learn package for each feature set and each preprocessing pipeline. The final accuracy was determined as the maximum accuracy among all machine learning methods.

**Results:**

No single optimal preprocessing pipeline for all feature vector construction algorithms was found. Smoothing increased accuracy for the voxel metrics ReHo and FHR, but not for the regional metric FCM. Spatial smoothing was the only preprocessing step that affected classification accuracy for the FHR method. The FHR feature set without smoothing demonstrated accuracy close to chance level. The steps of frequency filtering and median normalization substantially increased the accuracy for both the ReHo and FCM methods, both with and without smoothing. The steps of inhomogeneity correction and slice timing correction did not increase accuracy for the FCM feature set but did improve it by several percent for the ReHo feature set. Application of ICA filtering changed accuracy within a range of 5%, in either a positive or negative direction.

**Conclusion:**

Based on the obtained results, the following recommendations can be given for fMRI data preprocessing pipelines for binary classification of subjects into schizophrenia patients and healthy controls using machine learning methods and taking into account the feature vector construction algorithm. The most suitable for the ReHo metric pipeline includes motion correction, normalization, inhomogeneity correction, slice timing correction, frequency filtering, median normalization, and spatial smoothing. The most suitable for the FCM metric pipeline includes motion correction, normalization, inhomogeneity correction, slice timing correction, frequency filtering, and median normalization. Spatial smoothing is the most important factor for the FHR metric. The ICA filtering step does not provide a clear benefit and should therefore be applied with caution.

## Introduction

The functional magnetic resonance imaging (fMRI) has recently been used more often to analyze various factors affecting human cognitive processes, such as nicotine dependence, propensity for psychosis, alcoholism, hunger, etc. [1–4]. One of the up-to-date scientific fields is the study of the origin and course of mental disorders. Within this field, among other objectives, the classification of subjects based on the presence of mental disorders (in particular, schizophrenia) is being addressed using fMRI data and machine learning methods, such as support vector machines, random forests, etc. [5–7]. This objective is relevant as it is necessary to identify objective biological markers of these disorders.

To construct feature vectors in classification tasks, parameters based on correlation, signal frequency characteristics, graph metrics, etc., are often used. The values of these parameters strongly depend on the stage of raw fMRI data preparation, often referred to as data preprocessing, which includes steps to clean the data from hardware and physiological noise, as well as to standardize the data for group analysis (normalization, harmonization, etc.).

For example, Vergara et al. [1] studied the influence of specific preprocessing steps on the classification of subjects based on several features (mild traumatic brain injury, presence of harmful habits, e.g., smoking) using independent component analysis (ICA). The main focus was of the order of such two preprocessing steps as motion artifact correction using regression and ICA analysis. Additionally, the influence of the parameters of the frequency filtering step on the outcome was examined. The results showed that the highest accuracy was achieved by pipelines where the motion artifact correction step preceded the ICA analysis step. Notably, differences in accuracy associated with swapping the order of these two steps in the overall preprocessing pipeline reached 5–6%. Varying the upper threshold of frequency filtering showed a variability in accuracy within 2%.

The study by Gargouri [8] analyzed the impact of preprocessing steps on graph metrics, which are often used in fMRI-based classification tasks. The studied preprocessing pipelines included steps for phase shift correction, motion artifact correction, smoothing with a 5 mm Gaussian kernel, independent component filtering (tCompCor), and frequency filtering with thresholds of 0.01 and 0.1 Hz. Local and global efficiency, clustering coefficient, density, path length, among others, were selected as target graph metrics for analysis. Tests were conducted on healthy subjects. The choice and order of preprocessing steps was shown to affect graph metrics. Specifically, it was demonstrated that applying smoothing and tCompCor as the final steps in the preprocessing pipeline increased global efficiency.

The study by Candemir [9] analyzed the influence of spatial smoothing parameters (specifically, the smoothing kernel size) on fMRI data characteristics calculated using graph theory, as well as on the results of analysis using ICA and principal component analysis (PCA). The spatial smoothing kernel size was shown to affect node connections in the graph, leading to changes in functional network structures and PCA/ICA parameters. At the same time, graph metrics demonstrated greater stability to these changes.

Sotero et al. [10] studied the effect of frequency filtering steps and global signal regression on the accuracy of classification of subjects with autism spectrum disorders. Long short-term memory (LSTM) neural networks were used for classification. The absence of filtering was found to have higher classification accuracy, while no significant differences in classification accuracy were observed when the global signal was either removed or absent.

The work by Triana [11] demonstrated the impact of spatial smoothing on group differences between subjects diagnosed with autism spectrum disorders and bipolar disorder. Functional connectomes calculated on Brainnetome atlas regions were used as a feature set for group differentiation. Network-based statistics [12] was used to assess differences. The increase in the kernel size was shown to increase differences for functional connectomes where connection strength is expressed as a weighted matrix, while for functional connectomes with a thresholded connectivity matrix, it decreased differences. The authors concluded that spatial smoothing effects were difficult to predict, which should be considered when selecting a preprocessing pipeline.

Borchardt et al. [13] conducted a detailed study of the influence of three preprocessing steps (detrending, frequency filtering, removal of the global mean) on the separability of subjects with depression from healthy controls based on matrix and graph characteristics calculated from fMRI data. The authors concluded that preprocessing strongly affected subject separability. Particularly strong effects were observed from the removal of the global mean, the frequency filtering range, and the thresholding values used for sparsifying the connectivity matrix.

We did not manage to find studies investigating various preprocessing methods, pipelines, and algorithms in the context of binary classification of subjects into schizophrenia patients and healthy controls using machine learning methods. However, based on the cited works, preprocessing pipelines can be assumed to be a key factor for successful classification. Therefore, **the aim of this study** was to analyze the impact of various raw fMRI data preparation procedures on the accuracy of classifying subjects into schizophrenia patients and healthy controls using machine learning methods, and to give recommendations for optimizing the data preprocessing pipeline for this task.

We will further refer to “schizophrenia classification” meaning the binary classification task of distinguishing subjects with schizophrenia from healthy controls.

## Materials and Methods

### Brief overview of the method

**Figure 1.**
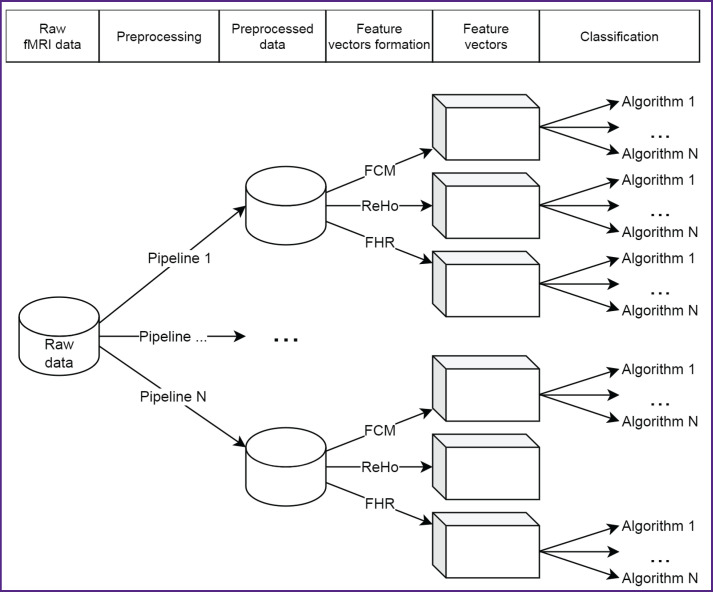
Research design

Figure 1 presents the general design of the experiment.

Raw data consisted of fMRI data from 72 subjects, acquired using a MRI scanner. For each dataset, several preprocessing variants were performed according to multiple examined preprocessing pipelines. In this study, 7 different preprocessing pipelines were analyzed. The basis was a classical fMRI data preprocessing pipeline consisting of 4 main steps.

Feature vectors for classification were constructed from each preprocessed dataset. We used 3 algorithms for feature vector construction: ReHo (regional homogeneity) [14], FCM (functional connectome matrix) [15], and FHR (functionally homogeneous regions) [16].

The classification was performed for each feature set and each preprocessing pipeline. In our work, subject classification was made using 15 machine learning methods from the scikit-learn package. The final accuracy was determined as the maximum accuracy achieved among all machine learning methods.

### Subjects

The study included 36 patients (16 women and 20 men; the mean age was 28.9±7.3 years) admitted to the general psychiatry departments of the Alekseev Psychiatric Clinical Hospital No.1 due to an exacerbation of productive symptoms of schizophrenia (diagnosis was verified according to the criteria of the F20 code of ICD-10) during the period 2018–2019. Patient examination and assessment were carried out within 48 h of admission to the 24-hour inpatient facility by two psychiatrists using a comprehensive approach. It included a clinical interview with the patient, a review of medical records, a patient examination, a collection of laboratory test results, an assessment of psychometric indicators and patient history, and interviews with relatives.

*Inclusion criteria* were the following: age range of 21–35 years; diagnosis of schizophrenia confirmed under the ICD-10; right-handed dominance; ability to critically assess one’s own condition; retention of memories of psychotic episodes; signed voluntary informed consent.

*Exclusion criteria* were the following: individuals with organic central nervous system (CNS) lesions, severe somatoneurological pathologies affecting brain structures, or a history of substance abuse. Additional exclusion factors included contraindications to MRI examination and refusal to participate.

The control group consisted of 36 healthy volunteers (17 women, 19 men; the mean age was 28.9±6.2 years) who were not related to the patients. All control participants underwent identical clinical and instrumental examination within a uniform protocol.

The study was conducted in accordance with the Declaration of Helsinki (2024) and was approved by the local ethics committee of the National Research Center “Kurchatov Institute” (protocol No. 5 dated April 5, 2017).

### Raw data collection

Experimental data were collected using a Siemens Magnetom Verio MRI scanner (Siemens Healthineers, Germany) with a magnetic field strength of 3 Tesla at the National Research Centre “Kurchatov Institute”. During the procedure, the subjects were instructed to close their eyes, relax as much as possible, and not think of anything specific. A multislice echo-planar imaging sequence was used to acquire fMRI data. Key parameters were the following: 56 slices, repetition time (TR) — 720 ms, echo time (TE) — 33 ms, field of view (FOV) — 192×192 mm, voxel size — 2×2×2 mm, multiband factor — 3. To obtain structural MRI, a T1-weighted sequence was used with the following parameters: 176 slices, TR — 1900 ms, TE — 2.19 ms, slice thickness — 1 mm, flip angle — 9°, inversion time — 900 ms, FOV — 250×218 mm. Additionally, to assess magnetic field inhomogeneity in the scanning area, a GRE field mapping protocol was used with the following parameters: 56 slices, TR — 470 ms, TE1 — 4.92 ms, TE2 — 7.38 ms, FOV — 192×192 mm, voxel size — 2×2×2 mm. In total, 900 time points of functional data were scanned, with a total duration of approximately 10.5 min.

### Data preprocessing pipelines

In this study, 7 different preprocessing pipelines were studied (14, considering the spatial smoothing step). The basis was a classical fMRI data preprocessing pipeline consisting of the following 4 main steps [17]:

1. calculation and correction of subject head motion during scanning using the BROCCOLI library [18] (5 iterations);
2. data correction for magnetic field inhomogeneity in the scanning area;
3. slice-timing correction (alignment to the signal acquired at a time point equal to half the TR value);
4. spatial normalization of individual images to the MNI (Montreal Neurological Institute) atlas space.

Steps 2–4, as well as the smoothing procedure (if applied), were performed using the freely available SPM12 software [19].

To improve the quality of the preprocessed data, a step to remove non-brain voxels was added between steps 1 and 2 of the pipeline described above. This procedure is based on probabilistic maps calculated from the data. The probabilistic map indicates the probability that a given voxel belongs to brain or non-brain tissue. The probabilistic map is usually constructed for the first fMRI image in the series. Then, by setting a probability threshold (usually 50%), this map is binarized and multiplied by each fMRI image. This approach requires complete correspondence of all voxels across the image series. This preprocessing pipeline is referred to as proc123.

It should be noted that steps 1 and 4 are mandatory for all preprocessing stages. Most fMRI studies are based on detecting small temporal changes in the BOLD signal caused by changes in blood oxygenation levels during brain activation. However, these changes are actually quite small (typically 1–5% even under optimal conditions and high magnetic field strength). Consequently, any head movement within 1 mm or rotation of 1 degree can lead to false activation estimates [20]. Moreover, all subsequent data processing steps require temporal stability of the spatial location of each voxel. Therefore, the motion correction step cannot be omitted in the present study.

Spatial normalization of data is a critically important step for whole-brain group analysis, allowing data to be averaged across all subjects. However, brain normalization is also essential for individual data, as it allows observed activated neuronal networks to be correlated with atlas regions in a standard spatial coordinate system. Brain normalization in volumetric space is typically performed by warping each individual brain image into a common space. After brain normalization, it is assumed that a point in the common space, defined by its coordinates (x, y, z), corresponds to an analogous region in any individual image normalized according to the same procedure. The most commonly used target spaces for normalization are the MNI space and the closely related Talairach space [21]. As data classification is performed considering regions from atlas space (based on the CONN atlas [22]), this step is also mandatory.

Thus, the preprocessing pipeline proc1 consists of applying only the 2 mandatory steps — motion correction and spatial normalization. The pipeline proc12 additionally includes a step for magnetic field inhomogeneity correction [23]. Unlike the pipeline proc12, the pipeline proc123 (the classical preprocessing option) includes an extra step of slice timing correction. The pipeline proc1234 additionally includes band-pass frequency filtering within the physiological range, as well as median intensity normalization of the dataset. The mean intensity calculated across all voxels is normalized to a specific value. This effectively removes any average global intensity differences between recordings from different subjects. Such a normalization procedure is highly relevant for fMRI data processing, as differences between datasets can be substantial. Performing it at the first (within-subject) level removes differences between subjects. Finally, pipeline proc12345 is obtained by using an ICA method to suppress noise from the data collected after applying pipeline proc1234. The ICA method is a powerful tool for blind source separation. In EEG, this method is widely used to detect and remove oculomotor artifacts. By analogy with EEG, ICA can be applied to fMRI data to detect and remove artifacts. As a result of applying ICA, fMRI data can be represented as three-dimensional spatial maps and corresponding time series [24]. Analysis of the spatial localization of a component and its temporal dynamics allows for the detection of noise components. To remove noise components, a neural network-based program developed by the authors was used.

Two additional data preprocessing pipelines appeared as a result of analyzing the accuracy of binary schizophrenia classification using the pipelines described above. It turned out that the steps of inhomogeneity correction and slice timing correction did not have a significant positive impact on accuracy. Therefore, the data were additionally processed using the following pipelines:

proc14 — motion correction, spatial normalization, frequency filtering, and median normalization;
proc145 — application of ICA to the data collected after processing with the proc14 pipeline.

Spatial normalization was applied as the final step in all the preprocessing pipelines described above.

Duplicate pipelines were also included for all the described data preprocessing pipelines, so the final step additionally contained spatial smoothing of the data using a Gaussian filter with a 6×6×6 mm kernel.

**Figure 2.**
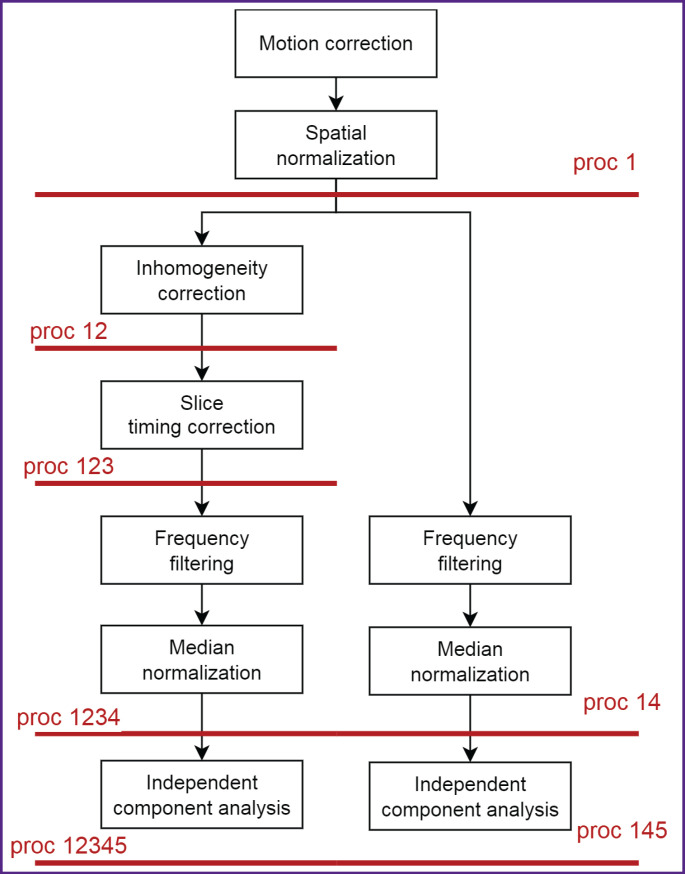
Studied fMRI data preprocessing pipelines For each pipeline, there is a duplicate version that finishes with a spatial smoothing step

For visual clarity, the data preprocessing pipelines and their brief descriptions are shown in Figure 2. Each pipeline ends with a spatial normalization step (not shown in the figure).

### Feature vector construction

In this study, we tested three algorithms for constructing feature vectors.

The regional homogeneity (ReHo) method [14] is based on calculating Kendall’s coefficient of concordance for each voxel with its nearest neighbors. In this study, we used 26 neighboring voxels. To reduce dimensionality, the resulting voxel metric was averaged over the regions of the CONN atlas. As a result, for each subject, the feature vector consisted of 132 values (corresponding to the number of regions in the CONN atlas). The BRANT software package v. 3.36 [25] was used to calculate ReHo values.

The method that forms correlation matrices of connectivity between brain regions is often referred to as the functional connectome matrix (FCM) [15]. The method is based on averaging voxel dynamics over the regions of an anatomical atlas (we used the CONN atlas), followed by calculating a correlation matrix between the resulting regional dynamics. Thus, for each subject, a 132×132 matrix is formed, but due to its symmetry, only the values above or below the main diagonal (a total of 132·131/2 = 8646 values) are used for classification. To calculate the FCM values, we used our own code. The correlation matrix was calculated based on Pearson’s correlation.

The previously developed functionally homogeneous regions (FHR) method [16] involves calculating the size of each voxel’s homogeneity region (i.e., the connected area around the voxel consisting of voxels that correlate with the given voxel above a specified threshold). As method’s results highly depend on data correlation, the following additional data preprocessing steps were performed for this method: voxel-by-voxel removal of autocorrelation using first- and second-order autoregression and removal of the global signal component through linear regression. In this study, the threshold was set to 0.5, as previous research [26] had shown that this threshold had achieved the highest classification accuracy. To reduce dimensionality, the resulting voxel metric was averaged over the regions of the CONN atlas, resulting in a vector of 132 values. The code from the GitHub website [27] was used to calculate FHR.

We chose the ReHo and FCM methods for analysis because they had proven effective in studies on automatic schizophrenia classification using fMRI data. At the same time, these methods differ substantially in scale: ReHo is a metric calculated at each individual voxel, whereas FCM operates at the scale of whole brain regions. We selected the FHR method because it had performed well in identifying a small number of the most informative features for schizophrenia classification [28]. Similar to the ReHo method, FHR is calculated at the individual voxel level.

### Classification

Subject classification was conducted using 15 machine learning methods from the scikit-learn package [29]: SVC (with RBF kernel), SVC (with linear kernel and increased regularization), NuSVC, LinearSVC, SGDClassifier, Perceptron, PassiveAggressiveClassifier, MLPClassifier, LogisticRegression, Logistic-RegressionCV, RandomForestClassifier, ExtraTrees-Classifier, RidgeClassifier, RidgeClassifierCV, and KNeighborsClassifier.

The dataset comprised only 72 subjects, that is why the accuracy for each machine learning methods was calculated using cross-validation based on repeated random subsampling (ShuffleSplit method) [30]. This method is used when there is a small amount of original data in order to obtain a more accurate result. At each iteration of the cross-validation loop, the 72 participants were randomly divided into a training set (64 samples) and a test set (8 samples). An important condition was that the test set had to contain samples from both classes. The classifier was trained on the training set, and its accuracy was then calculated on the test set. We used 1000 iterations of the cross-validation loop for each data processing variant, calculating the resulting accuracy according to the following formula:

Accuracy=1nΣi=1nTNi+TPiTPi+TNi+FPi+FNi,

where *n* is the number of iterations (in our case, 1000); *TP* and *TN* are the number of true positive and true negative results in each cross-validation iteration, respectively; *FN* and *FP* are the numbers of false negative and false positive results in each cross-validation iteration, respectively. The index *i* denotes the iteration number.

The final accuracy was determined as the maximum accuracy among all machine learning methods.

## Results

The results of the influence of fMRI data preprocessing on the accuracy of schizophrenia classification using machine learning methods are shown in Tables 1 and 2.

**Table 1.** Influence of fMRI data preprocessing on schizophrenia classification accuracy using machine learning methods (without smoothing)

Preprocessing pipeline	ReHo	FCM	FHR
proc1	0.64	0.64	0.62
proc12	0.58	0.65	0.54
proc123	0.59	0.64	0.52
proc1234	0.68	0.81	0.55
proc14	0.65	0.77	0.64
proc12345	0.64	0.77	0.61
proc145	0.62	0.79	0.63

**Table 2.** Influence of fMRI data preprocessing on schizophrenia classification accuracy using machine learning methods (with smoothing)

Preprocessing pipeline	ReHo	FCM	FHR
proc1	0.64	0.65	0.74
proc12	0.62	0.64	0.74
proc123	0.61	0.65	0.71
proc1234	0.76	0.78	0.72
proc14	0.73	0.80	0.75
proc12345	0.73	0.80	0.72
proc145	0.75	0.81	0.78

The following conclusions can be drawn from the obtained results.

During the binary schizophrenia classification using resting-state fMRI data with machine learning methods, the choice of preprocessing pipeline can change the classification accuracy by more than 20% (depending on the feature vector construction method). In particular, for the ReHo method, the minimum accuracy was 58%, while the maximum was 76%; the accuracy of the FCM method ranged from 64 to 81%; and for the FHR method — from 52 to 78%.

There is no single optimal preprocessing pipeline for all feature vector construction algorithms (FCM, ReHo, FHR).

Smoothing increased accuracy for the voxel metrics ReHo and FHR (in some cases by more than 10%), while there was no such a significant improvement for the regional metric FCM (maximum difference 3%). The reason for this might be connected to the fact that smoothing reduces noise in individual voxels at the voxel level. As for calculation of the regional metric FCM, the data are initially averaged over regions, which is equivalent to smoothing in voxel metrics.

For the FHR method, only spatial smoothing significantly affected classification accuracy among all the preprocessing steps. The FHR feature set without smoothing was characterized by the accuracy close to chance-level.

Step 4 (frequency filtering and median normalization) provided a substantial increase in accuracy for both the ReHo and FCM methods, both with and without smoothing. The maximum increase showed that the FCM was 17% when switching from the proc123 pipeline to the proc1234 pipeline in the option without smoothing.

Comparing the accuracy of the FCM and ReHo pipelines that included steps 2 (field inhomogeneity correction) and 3 (slice timing correction) with similar pipelines without these steps, it can be concluded that steps 2 and 3 do not consistently increase classification accuracy: the accuracy change from adding these steps ranged from –5% to +3%.

The application of ICA filtering (step 5) changed accuracy within a range of 5% but could work in either direction (improving or reducing accuracy), which makes it difficult to determine whether it is necessary.

## Discussion

Over the past decade, a large number of studies [1, 8, 9, 11–13] have been published describing the processing of fMRI time series. A huge number of preprocessing steps has been tested, the minimally necessary steps have been identified, and their optimal order within the overall processing pipeline has been determined. However, the obtained findings were based on analysis of the quality of the resulting data, as well as on the results of statistical mapping performed after preprocessing. In the present study, we hypothesized and tested that the most optimal data preprocessing pipeline in terms of quality metrics and statistical analysis may not provide the best accuracy for the binary schizophrenia classification. Overall, it can be noted that preprocessing using the classical pipeline (referred to as proc123 in this article) did not achieve an accuracy above 65% for any of the feature metrics examined. Preprocessing using band-pass filtering and intensity normalization showed the best results. The importance of the frequency filtering step is consistent with the findings [13] showing that the correct choice of filtering threshold significantly improves the separability of a clinical depression group from a control group when using matrix and graph characteristics calculated from FCM.

Thus, the set of preprocessing steps and their order should be determined by the specific task, and the set of methods implementing the selected steps, along with their parameters, can be a subject of research.

The FHR method deserves special mention, as its accuracy depends predominantly only on the spatial smoothing step. Comparing the accuracies in Table 2, it can be noted that the FHR method outperforms all other methods prior to the application of frequency filtering and median normalization. Even after applying these steps, the accuracy achieved on the FHR feature set is not significantly lower than that achieved on the other feature sets. This is important because it allows new data to be quickly introduced into the analysis, whereas a full preprocessing cycle (including steps 4 and 5) is time-consuming and, in some cases, requires manual expert intervention. At the same time, spatial smoothing does not require a substantial time investment.

This research has several limitations. First, the study was conducted using fMRI data acquired from a single scanner using a single scanning protocol. However, recent studies have shown that the accuracy of classifying cognitive disorders using fMRI data can depend on the specific dataset [31], i.e., on both the used scanner and the scanning parameters. It is logical to assume that preprocessing may also have different effects on different datasets.

Second, this study tested only a few pipelines built upon the classical fMRI data preprocessing pipeline [17]. Consequently, a number of preprocessing algorithms and methods (e.g., global signal regression) remain outside the scope of this article.

Third, while this study combined various data preprocessing steps, it did not examine the parameters of these steps. For example, we used spatial smoothing with a 6 mm Gaussian kernel, without investigating other kernel sizes.

As a direction for future research, we propose fixing a data preprocessing pipeline and optimizing the parameters of the steps included in the chosen pipeline.

## Conclusion

For binary schizophrenia classification using resting-state fMRI data with machine learning methods, the choice of preprocessing pipeline can change the classification accuracy by more than 20%, depending on the feature vector construction method.

There is no single optimal data preprocessing pipeline for different feature vector construction algorithms.

Based on the obtained results, the following recommendations can be given for fMRI data preprocessing pipelines for binary schizophrenia classification, taking into account the feature vector construction algorithm.

The most suitable for the ReHo metric pipeline includes motion correction, normalization, inhomogeneity correction, slice timing correction, frequency filtering, median normalization, and spatial smoothing.

The most suitable for the FCM metric pipeline includes motion correction, normalization, inhomogeneity correction, slice timing correction, frequency filtering, and median normalization.

Spatial smoothing is the most important factor for the FHR metric.

The ICA filtering step does not provide a clear benefit and should therefore be applied with caution.
